# Case of Arachnitis

**Published:** 1842-07-16

**Authors:** 


*Case of Arachnitis, with suppression of the Urine for three days.*

J. P., a coloured man, ætat. 21, entered the hospital May the 29th. No satisfactory information could be obtained of the early part of his illness. He said he had been sick for about nine days, and was first taken with cephalalgia, which was followed at the end of a day or two by general pain, not limited to any place, and continued until his entrance.

When he entered the ward he offered the following symptoms: Expression anxious; eyes suffused; conjunctiva injected; dyspnœa; intellect dull; no delirium; tongue large and white, with elevated papillæ; skin dry, and moderately warm. Pulse 98, feeble; bowels constipated; extreme cutaneous sensitiveness; complains of pain on moving; great prostration. The physical signs indicated but little disease of the chest; the respiratory murmur was feeble in the lower lobe of the right lung, with slight traces of crepitus. Six scarified cups to be applied on the right side of the chest, and a dose of oil.

30th. Patient makes frequent complaints; respiration sighing, complaining; head slightly elevated; no cephalalgia; no tinnitus; hearing is good; thirst moderate; slight subsultus; almost complete anorexia. Pulse 80; skin cool. He has no cough; no expectoration. Six scarified cups were applied between the shoulders along the spine.

℞. Pulv. Ipecac. et Opii, } *aa.* gr. xxvj.

Camphoræ, }

Syrupi, q. s.

M. Et. ft. Mass. in pil. xij. Div.

Take one every three hours.

31st. Expression stupid; vomited frequently during the night; slept none. Pulse 72, small and feeble; respiration 24. The anodyne was continued, and five grains massa hydrargiri, with a few grains of soda bicarbonas, were directed to be given three times a day.

June 1st. Stupidity continues, but there is no positive delirium; cutaneous sensitiveness continues; his movements are painful and difficult; constant moaning and crying. He has passed no urine for the last twenty-four hours. A blister was applied to the spine, and five grains of the carbonate of ammonia given every two hours. The mercurial and the anodyne pills were continued.

2d. Extreme prostration; stupor increased; he neither eats or drinks; passes no urine. A catheter was introduced in the bladder, but no urine flowed from it. He was directed a turpentine clyster. He died during the night.

*Necroscopy twelve hours after Death.*

The arachnoid membrane on the upper surface of the brain was opaque, and presented spots of bright red injection, stellated. The substance of the brain was natural, and there was no fluid in the ventricles. The membranes of the spinal cord were thickened, and the substance of the cord unusually injected.

The pleura costalis and pulmonalis were adherent on the right side, and the lower lobe of the right lung was reddened and congested. The other viscera of the thorax were healthy.

The *kidneys* were excessively enlarged—nearly twice the usual size. Externally they were of a dark red colour, and mottled. In the corticle substance the granular degeneration was very evident, more particularly between the tubes, where the yellowish tint of the granulations predominated over the darker colour, which was seen nearer the surface. The bladder contained about a pint of straw coloured fluid.

